# Investigation on the Distribution of *Bangana Tungting* in Yuanshui Unique Fish Species National Aquatic Germplasm Resources Reserve Using Environmental DNA Technology

**DOI:** 10.1002/ece3.70626

**Published:** 2024-12-12

**Authors:** Lu Tian, Qianqian Wu, Li Zou, Jinxin Zhou, Chongrui Wang, Linmei Han, Zaiquan Zhang, Xing Xiang, Mingqiu Liu, Zhifeng Feng, Zhonggui Xie, Zhiqiang Liang

**Affiliations:** ^1^ Hunan Fisheries Science Institute Changsha Hunan China; ^2^ Aquatic Products Seed Stock Station in Hunan Province Changsha Hunan China; ^3^ Graduate School of Human Development and Environment Kobe University Kobe Japan; ^4^ Institute of Industrial Science The University of Tokyo Kashiwa Japan; ^5^ Huaihua Tianling Nature Protection Company Huaihua Hunan China; ^6^ Animal Husbandry and Aquatic Products Center of Hongjiang District Huaihua Hunan China

**Keywords:** *Bangana tungting*, dissolved oxygen, distribution, environmental DNA, hydroelectric station construction, reserve

## Abstract

Freshwater ecosystems face unprecedented challenges as the cumulative impact of human activities intensifies. While protected areas and species‐specific conservation policies are widely implemented, their effectiveness remains difficult to gauge using traditional catch‐based surveys. This research employed environmental DNA (eDNA) technology to assess the distribution of the endangered fish, *Bangana tungting*, within the Yuanshui Unique Fish Species National Aquatic Germplasm Resources Reserve (YUFRR) in Hunan, China. Over a 2‐year period, we conducted comprehensive eDNA survey multiple sites within the YUFRR, confirming the species' continued existence in the area. In September 2022, *B. tungting* eDNA was detected at 8 of 60 sampling locations, while a follow‐up survey in May 2023 identified its presence at 4 of 44 sites. Further analysis revealed critical environmental factors influencing *B. tungting* distribution, primarily dissolved oxygen concentration and the presence of physical barriers such as hydroelectric stations. Our data suggest a minimum dissolved oxygen tolerance threshold of 4 mg/L for this species. Moreover, we observed an inverse relationship between *B. tungting* detection rates and both the number of hydroelectric stations and their distance to sampling sites. This case study demonstrates the effectiveness of eDNA technology in mapping the distribution of endangered fish species like *B. tungting* and guiding conservation strategies. Our findings emphasize the crucial need to enhance environmental conditions, particularly water quality and habitat suitability, to ensure the effective conservation of *B. tungting* within the YUFRR.

## Graphical abstract

A 2‐year survey, using environmental DNA technology, was conducted to detect the presence of *Bangana tungting* in the Yuanshui Unique Fish Species National Aquatic Germplasm Resources Reserve (YUFRR), Hunan, China.

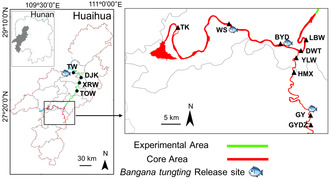

## 1 Introduction

The alarming decline in freshwater species diversity has attracted widespread attention. The IUCN's (International Union for Conservation of Nature and Natural Resources) latest update completes the first comprehensive assessment of freshwater fish species, revealing that 3086 out of 14,898 assessed species (25%) face extinction (IUCN 2023). This phenomenon is caused by several factors, including global climate change (Aziz et al. 2021; Barbarossa et al. 2021); human activities (Su et al. 2021)—in particular, river ecosystems have been damaged by the overexploitation of rivers and hydroelectric station construction for hydroelectric power (Barbarossa et al. 2020); and the invasion of non‐native species, often resulting in predation on small fish, the habitat shrinkage of native fish species (Özdilek, Partal, and Jones 2019; Lusk, Lusková, and Hanel 2010; Strayer 2010), and eventually a severe loss of diversity and biomass (Castaldelli et al. 2013; Wang et al. 2021). Take China as an example, and in the third assessment of China's Red List of freshwater fishes, it was found that 22.3% of freshwater fish species are under threat (Cao et al. 2023). In recent years, China has established numerous freshwater fish sanctuaries and implemented various conservation policies (such as fishing bans and restocking programs; Wang, Yu, and Hu 2024; Zheng et al. 2012). However, the effectiveness of these measures in the areas still requires further evaluation.

**FIGURE 1:**
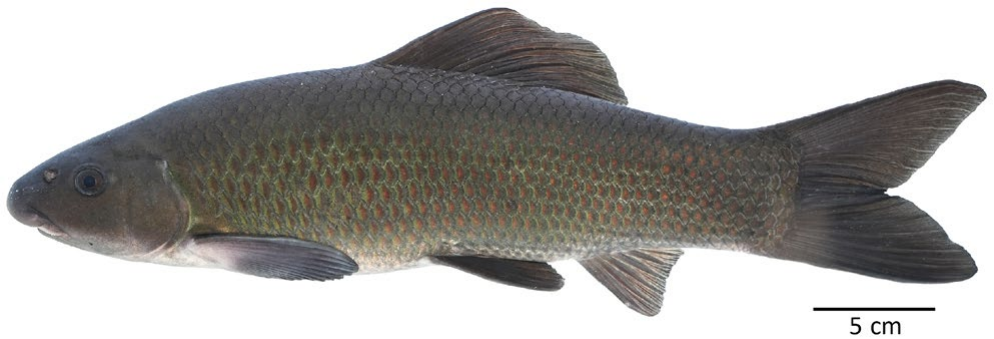
The focus of this research is on the target species, *Bangana tungting*, which is a large indigenous fish species endemic to China.

*Bangana tungting* (Nichols 1925; Figure 1) is classified as a vulnerable species according to both The Red List and FishBase (http://www.fishbase.org). The large indigenous fish species unique to China had an annual production exceeding 160 tons in the 1940s and 1950s (Bian et al. 2011). Field surveys from 2006 to 2010 indicate that due to overfishing and river development, the population of *B. tungting* has drastically declined in its historical distribution areas, including the Xiangjiang, Zijiang, Yuanshui River, and Lishui River basins and Dongting Lake (Bian et al. 2011). Currently, the species is mainly found in the Yuanshui Unique Fish Species National Aquatic Germplasm Resources Reserve (YUFRR) and is considered endangered (Bian et al. 2011). To protect and restore the wild populations of this species, Hunan Province has been conducting conservation research on *B. tungting*. In 2010, the YUFRR was established in Huaihua City with a comprehensive “no‐fishing” policy being introduced (Yin et al. 2022) and includes two functional areas (experiment area and core area) according to Chinese regulations. Significant advancements have been made in artificial breeding and large‐scale propagation techniques, resulting in the breeding of 8.65 million juvenile *B. tungting* from 2007 to 2021 (Li et al. 2022). The local government had set up several sites for fish releasing, in which *B. tungting* has annually been released into rivers since 2009. Currently, occasional sightings of *B. tungting* near the release sites suggest a potential recovery of the species; however, delicate investigation has not been conducted yet. To scientifically assess the effectiveness of the releases and enhance the efficiency of future efforts (e.g., optimally selecting release sites and quantities), there is an urgent need to develop a simple and highly sensitive survey method to ascertain the current status of *B. tungting* resources.

Traditional fisheries resource surveys are preferred regarding the fundamental data such as relative abundance, biomass, age structure and maturity of fish populations (Beentjes and Carbines 2012; Maunder and Punt 2013). From a technical perspective, traditional methods for identifying fish species rely on qualitative and quantitative characteristics of specimens (Thomsen et al. 2016). Meanwhile, traditional ichthyological classification methods are often constrained by topography, sampling tools, seasonal variations, and the availability of specialized technical knowledge. In addition, the unique traits and habits of *B. tungting* pose challenges to conventional survey methods. For instance, its elongated body resembles that of the *Mylopharyngodon piceus* , causing difficulties in traditional fisheries resource surveys based on morphology (Wang, Liu, and Tian 1982). These factors make it difficult for traditional methods to capture or track the movements of *B. tungting* populations, thereby complicating the study of their resource dynamics.

In recent years, environmental DNA (eDNA) technology has emerged as a novel method for monitoring aquatic biodiversity. It enables the extraction of DNA, originating from biological remnants like skin tissues and gametes, from environmental samples such as water and sediments, providing insights into the biological resources within habitats (Lacoursière‐Roussel et al. 2016). Compared to traditional fishery resource surveys, eDNA sampling offers minimal ecological disturbance, ease of operation, and high sensitivity and is less affected by location and seasonal constraints, making it particularly suitable for monitoring rare species (Gehri et al. 2021; Hallam et al. 2021; Li et al. 2020). The presence of species is determined through the collection of water sample, followed by DNA extraction and PCR amplification using specific primers and probes. This approach is widely utilized in freshwater ecological surveys (Doi et al. 2017; Pont et al. 2018; Spear et al. 2021; Wu et al. 2023; Pont et al. 2023), demonstrating that the applicability of eDNA technology is feasible in the research of *B. tungting* in river systems.

In this study, we developed a DNA‐based method specifically for investigating the distribution of *B. tungting* resources in rivers, utilizing membrane filtration for DNA enrichment and real‐time PCR amplification. To achieve these goals, we first developed specific primers for *B. tungting* and conducted specificity tests on these primers. We then analyzed eDNA samples from the field in the YUFRR to ascertain the distribution of *B. tungting* and examine the impact of environmental factors on the detection rates of this species.

## 2 Materials and Methods

### 2.1 Primer Development

We developed species‐specific primers and probes for the detection of *B. tungting*, which do not amplify DNA from other species within the same genus. Due to its high mutation rate, abundance in cells, and higher coverage in genetic databases, mitochondrial DNA (mtDNA) is used mostly as a genetic marker (Goldberg et al. 2016; Handley 2015). In environmental DNA (eDNA) studies, primers targeting mitochondrial genes such as cytochrome c oxidase subunit I (COI), D‐loop, 12S ribosomal RNA (12S), and cytochrome b (Cytb) are frequently employed for the detection of specific species (Tsuji et al. 2019). This study focused on the Cytb gene because corresponding sequences are publicly available not only for the target species but also for closely related species within public databases at the National Center for Biotechnology Information (NCBI). Initially, the mtDNA sequence of the Cytb of *B. tungting* and eight sequences from nontarget species within the same genus were obtained from GenBank (https://www.ncbi.nlm.nih.gov/genbank/) (Table S1). Primer Express (version 3.0; Applied Biosystems, Foster City, California) was used to design a set of species‐specific primers and a TaqMan probe with default settings. To ensure that the designed primers would not amplify DNA from related nontarget species, a Basic Local Alignment Search Tool (BLAST) search was conducted using default settings to identify potential amplification targets. Additionally, total DNA was extracted from the caudal fins of both target and nontarget species (Table S1) using the DNeasy Blood and Tissue Kit (Qiagen, Hilden, Germany), and PCR amplification was performed with a concentration of 1 pg/μL. Subsequently, the sequences were confirmed by the direct sequencing of successfully amplified PCR products, using DNA from both target and nontarget species as templates. This was carried out by a commercial service to confirm the sequences (Shanghai Shenggong Biological Engineering Co. Ltd., China).

### 2.2 Sampling Area

**FIGURE 2:**
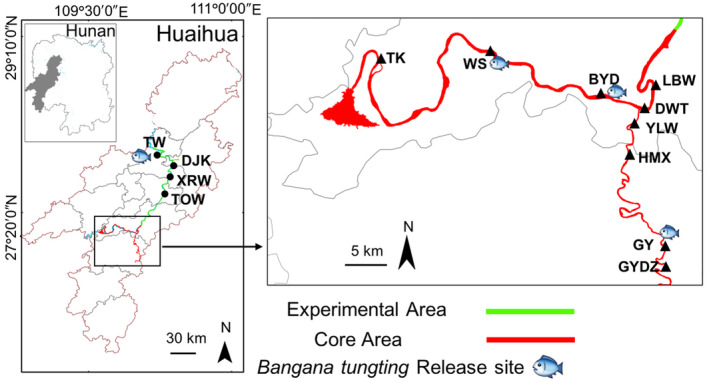
Distribution map of sampling areas in the Yuanshui Unique Fish Species National Aquatic Germplasm Resources Reserve (YUFRR).

The YUFRR is located in Huaihua City, Hunan Province. Its protection scope is mainly river waters. In September 2022 (autumn) and May 2023 (summer), we collected water samples from 13 sampling areas, comprising of 60 and 44 sites in the YUFRR, respectively (Figure 2; Table S2). However, 16 sites were unable to be sampled in 2023 due to climatic reasons. The sampling areas were located along the main channel of the Yuanshui River and its tributary, the Wushui River. Some areas, such as TK, GY, YLW, GYDZ, DWT, and BYD, are located in the core area, while the TOW, TW, DJK, and XRW are situated in the experimental area (Figure 2; Table S2). The core area is strictly restricted, with limited personnel access. The experimental area is allowed to conduct activities and explore nature protection under the condition of not affecting the protection work. The level of protection in the core area is higher than that in the experimental area. Notably, *B. tungting* was released in the BYD river section for the past 2 years (Table S2). Water samples collected from the site represented a regional composite. Each sampling site was spaced at least 1 km apart to collect water samples, with 1 L of surface water collected at each site. To prevent DNA degradation in the samples, 1 mL of BCA (benzalkonium chloride solution) was added to the collected water samples (Yamanaka et al. 2017). During the sampling process, gloves were changed after each sample was collected, and the entire procedure was conducted according to the standards set by the Japan eDNA Society (2019). Disposable bottles were used for water sample collection. Each day of sampling included a field control sample, consisting of a 1‐L bottle of mineral water, and the bottle was opened in the field and treated with 1 mL of BCA. During the September 2022 sampling, water temperature, dissolved oxygen, ammonia nitrogen, and pH were simultaneously measured at each site using a YSI ProQuatro multiparameter meter (Yellow Springs, Ohio, USA). Due to adverse weather conditions, the environmental factors were not assessed in May 2023. This field investigation was conducted with the approval of the Animal Husbandry and Aquatic Products Center of Huaihua, China, under application number XH2023016. All procedures were performed in accordance with relevant guidelines and regulations, and informed consent was obtained from all participants involved.

### 2.3 Treatment of Water Samples and DNA Extraction

All water samples were vacuum‐filtered through a one glass‐fiber filter with a nominal pore size of 0.7 μm (GF/F; Shanghai Bitai Biotechnology Co. Ltd., Shanghai, China). The filtration of samples was completed on the day of water collection. Filters were stored in a 2‐mL Eppendorf tube and preserved on dry ice during the field sampling period and subsequently placed in a laboratory freezer at −30°C until DNA extraction. Filtration equipment was soaked in a 10% sodium hypochlorite solution for at least 5 min before and after use to remove residual DNA (Wu et al. 2018), then thoroughly rinsed with tap water, followed by a final rinse with commercially available purified water (Hangzhou Wahaha Group Co. Ltd., China). Disposable gloves were used for each sample during the filtration process.

In this study, total DNA was extracted from the filters using the DNeasy Blood and Tissue Kit (Qiagen, Hilden, Germany). The specific methods and volumes of reagents used for DNA extraction were modified according to Wu et al. (2023), including the use of 440 μL of ATL, 400 μL of AL, and 40 μL of ProK. After extraction, the total DNA was eluted with 110 μL of AE (Qiagen, Hilden, Germany) and stored at −20°C (Haier, FCD‐241LHSC) until PCR amplification.

### 2.4 PCR Amplification

The real‐time TaqMan PCR with the LightCycler 96 System (Roche Diagnostics) was utilized to identify the presence of *B. tungting* DNA in field water samples. The real‐time PCR was carried out in a 20‐μL reaction volume, containing 900 nM of each primer, 125 nM of TaqMan probe, 1 × Environmental Master Mix 2.0 (Life Technologies), 0.1 μL of AmpErase Uracil N‐Glycosylase (Thermo Fisher Scientific), and 2 μL of template DNA. The qPCR conditions were as follows: initial denaturation at 95°C for 10 min, followed by 55 cycles of 95°C for 15 s and 60°C for 1 min, with an initial hold at 50°C for 2 min. Each sample was analyzed in triplicate, and ultrapure water replaced the template DNA in negative controls on all PCR plates. In this study, a sample was considered positive if any of the triplicate reactions yielded an amplification result (Wu et al. 2023). Finally, we conducted sequencing of the amplified PCR products and compared the resulting sequences as the target species sequences using the BLAST service provided by the NCBI (https://blast.ncbi.nlm.nih.gov/Blast.cgi). A positive result was defined as a sequence exhibiting complete consistency.

### 2.5 Data Analysis

To assess the potential impact of hydroelectric stations on the distribution of biota, the number of densely situated hydroelectric stations at each sampling point was determined using Google Maps. Additionally, we utilized Google Map's scaling tools to calculate the shortest straight‐line distances between each sampling site and the nearest hydroelectric station. Following this, a selection process was conducted on the environmental data collected. We computed pairwise Pearson correlation coefficients (*r*) to examine collinearity among the environmental factors collected in September 2022. When two or more environmental factors were highly correlated (i.e., |*r*| > 0.7), only one factor was retained (Dormann et al. 2013). Using the filtered environmental factors, we qualitatively examined the relationship between the presence of biota and various environmental factors.

## 3 Results

### 3.1 Effectiveness of the Primers

**FIGURE 3:**
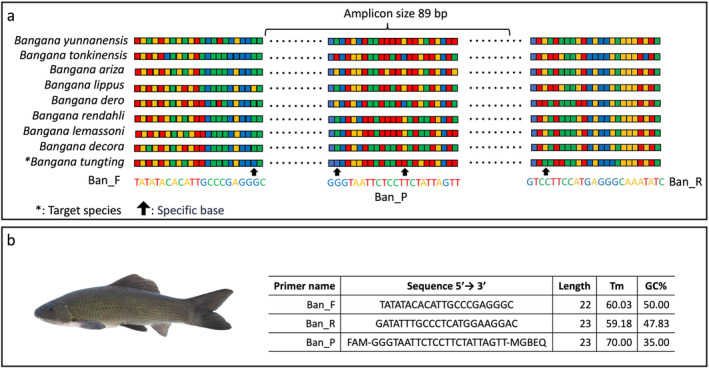
Primer sequences developed in this study: (a) the base sequences of target and nontarget species and the positions of primers developed in this study and (b) the sequence, length, *T*_m_ value, and GC% of the developed primers.

We designed specific primers and probes for the *B. tungting* in the Cytb gene (Figure 3). To verify the specificity of the primers developed in this study, a BLAST search of the primers indicated that they do not amplify species from the genus *Bangana* other than target species. Additionally, we successfully amplified the Cytb gene of *B. tungting* from tissue samples. Importantly, there was no cross‐reactivity with commonly related species. The PCR sequencing of the amplified products revealed sequences corresponding to the target species (Figure S1).

### 3.2 *Bangana tungting* eDNA in Field Sampling

In September 2022, eDNA of *B. tungting* was detected at 8 of 60 sampling sites. These detections occurred in the TK, BYD, XRW, GY, and YLW sampling areas (Table S2). Specifically, *B. tungting* eDNA was identified at one site in each of the first three areas (TK, BYD, XRW) and at three and two sites in the latter two areas, respectively (Table S2). In a subsequent survey conducted in May 2023, *B. tungting* DNA was found at 4 of 44 sampling sites, specifically at TK, BYD, DWT, and GYDZ (Table S2). The cycle threshold (*C*_t_) values at the sites detected in 2022 ranged from 36.49 to 39.09, with a detection rate between 0% and 41.7%; in 2023, and the *C*_t_ values ranged from 35.96 to 49.13, with a detection rate between 0% and 25% (Table 1). In this research, no target species DNA was found in any of the negative controls, including field controls.

**TABLE 1:** Real‐time PCR positive results and detection rates across sampling areas in September 2022 and May 2023. We assessed the number of positive cases across survey areas and sampling months and the detection rates of positive cases in monthly samples.

Sampling area	September 2022		May 2023	
	Number of positive	Detection rate	Number of positive	Detection rate
TK	1	4.2%	1	4.2%
WS	0	0.0%	0	0.0%
BYD	1	8.3%	1	8.3%
DWT	0	0.0%	1	25.0%
LPW	0	0.0%	0	0.0%
TongW	0	0.0%	Missing data	Missing data
XRW	1	8.3%		
DJK	0	0.0%		
TW	0	0.0%		
GYDZ	0	0.0%	1	8.3%
GY	1	12.5%	0	0.0%
HMX	0	0.0%	0	0.0%
YLW	2	41.7%	0	0.0%

### 3.3 Relationship Between the Distribution and Environment

**FIGURE 4:**
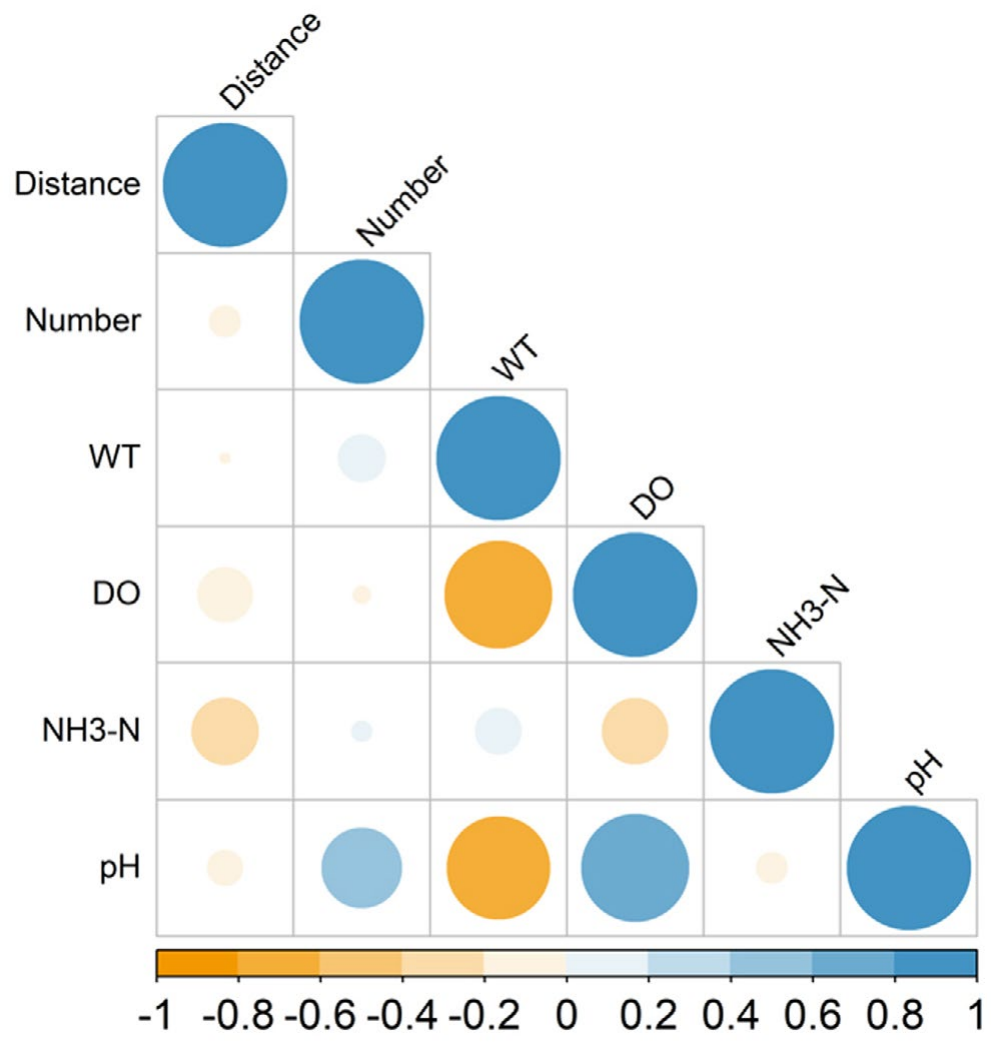
Correlation between the number of and distance from hydropower stations, ammonia nitrogen (NH_3_–N), water temperature, dissolved oxygen concentrations, pH, and detection of *Bangana tungting* eDNA. This figure is based on the monitoring data in September 2022.

We initially observed that environmental variables could be broadly categorized into two groups: Group 1 includes the number of hydropower stations, their proximity, and ammonia nitrogen (NH3–N); Group 2 comprises water temperature, dissolved oxygen concentration, and pH values. Variables in Group 1 generally exhibit low correlations among each other, whereas those in Group 2 are highly correlated. Notably, water temperature and dissolved oxygen show a strong correlation (|*r*| = 0.75, *p* < 0.001; *N* = 56, *t* = −8.26), and there is also a significant correlation between dissolved oxygen and pH (|*r*| = 0.75, *p* < 0.001; *N* = 56, *t* = −8.32) (Figure 4). Consequently, in our subsequent analyses, we retained all variables from Group 1, and from Group 2, we selected dissolved oxygen concentration due to its statistical importance (Table S3), susceptibility to natural disturbances (Chen et al. 2002), and the possible role in conservation strategies.

**FIGURE 5:**
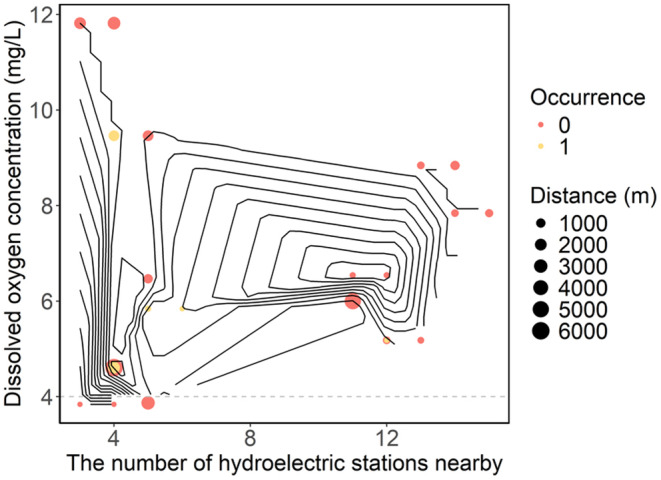
Relationship between *Bangana tungting* eDNA presence and dissolved oxygen, ammonia nitrogen, the number of, and distance from hydropower stations. Red circles indicate no detection of *B. tungting* eDNA, and yellow circles indicate detection. The size of the circles represents the distance between the sampling points and the nearest hydropower station. The contour lines show the ammonia nitrogen concentration. The gray dashed line represents the lower acceptance limit of the target species for dissolved oxygen.

We employed a qualitative comparative approach to explore the relationships between the presence of *B. tungting* eDNA (indicated by dot colors) and various environmental factors such as dissolved oxygen concentrations, the number of hydropower stations (*x*‐axis), distance from these stations (dot size), and ammonia nitrogen (NH_3_–N) concentrations (indicated by contour lines). Initially, dissolved oxygen concentration emerges as the most evident environmental factor. Despite a relatively wide range of dissolved oxygen concentrations at points of detection, *B. tungting* eDNA is no longer detectable when dissolved oxygen falls below 4 mg/L (Figure 5). Based on this observation, it is hypothesized that the minimum tolerance limit for *B. tungting* in terms of dissolved oxygen is 4 mg/L. Moreover, the detection rate of *B. tungting* also appears to be influenced by the number of nearby hydroelectric stations and their proximity to the survey sites (Figure 5). The probability of detection varies with an increase in the number of hydroelectric stations and their distance from these sites. Generally, the closer the sampling point is to a hydroelectric station, the more likely it is to detect the target species. Conversely, the presence of a higher number of hydropower stations in the vicinity of a sampling point tends to decrease the likelihood of detection. However, considering both the number of stations and their distances, we speculate that certain combinations might result in a higher occurrence rate (e.g., having four to five hydroelectric stations located between 1000 and 3000 m away; Figure 5). The NH_3_–N concentrations at sites where the target species were detected in September 2022 were relatively uniform, ranging from 0.04 to 0.09 mg/L (Figure 5; Table S2).

## 4 Discussion

This study has successfully developed a detection method for the DNA research of *B. tungting*, marking a significant breakthrough in conservation research for this species. Although the detection rate of this species in the wild is not high, we have successfully detected *B. tungting* eDNA at several sampling sites in YUFRR. This detection provides evidence of the presence of this species in the area and suggests that eDNA technology may be applied to assess the effects of restocking efforts. Through the analysis of the relationship between biological distribution and environmental factors, we found that the presence of *B. tungting* is influenced by various environmental factors, including dissolved oxygen concentrations and physical barriers such as hydroelectric stations. Therefore, improving and adjusting these environmental factors are crucial for the conservation of *B. tungting*. Our findings have identified the most favorable and unfavorable environmental conditions for the survival of *B. tungting*, providing foundational insights for future studies and the delineation of management areas for this species.

### 4.1 *Bangana tungting* eDNA Results in Field Sampling

Following the successful development of large‐scale breeding techniques for *B. tungting*, the annual release of artificially bred *B. tungting* juveniles has steadily increased (Department of Agriculture and Rural Affairs of Human Province 2023). We conducted a 2‐year survey using eDNA technology to investigate the presence of *B. tungting* eDNA in the YUFRR (Table 1). In this research, more than two out of three replicates showed positive results with significant *C*_t_ values (Table S2), and DNA concentration in a sample can be indirectly determined by the *C*_t_ value obtained from PCR (Kusanke et al. 2020). Specifically, higher DNA concentrations tend to have lower *C*_t_ values with a higher rate of reproducibility among three replicates, and vice versa. Thus, it is possible to postulate high concentrations of target species' DNA. Notably, in the YLW area during the survey in September 2022, YLW2 and YLW4 sites revealed considerable detection rates of *B. tungting* eDNA (Table S2), suggesting a large population in this area.

One out of the total 12 sites where the species were detected is located within the experimental area of the YUFRR (XRW river; Table S2). The remaining 11 sites were found within the core area (TK, BYD, DWT, YLW, GY, GYDE river; Table S2). Historical records indicate that the YUFRR was once a significant habitat for *B. tungting* (Li et al. 2022). Notably, *B. tungting* was released in the BYD river section for the past 2 years (Yueyang Municipal People's Government 2023). However, due to factors such as hydroelectric station construction and overfishing, its population has nearly vanished from the most waters, except for only a small portion of the Yuanshui River. We observed that some positive sites were located in unreleased areas, while others were located in released areas. According to the available data, *B. tungting*'s survival and reproduction may be influenced by the total number of hydropower stations, expect for the XRW area. Other factors, such as abundant vegetation and food resources, may also influence the survival and reproduction of *B. tungting*. Moreover, as *B. tungting* is detritivores (Liang et al. 2011), the further detailed investigation of the relationship between environment and the detection of *B. tungting* is necessary.

### 4.2 Environmental Factor Analysis

This study's assessment of environmental factors, water quality, in particular, is based on survey results in September 2022. Although there is a degree of randomness, the findings still reveal fundamental relationships, such as a high correlation between water temperature and dissolved oxygen concentration (Figure 4). Additionally, this analysis incorporates anthropogenic factors, such as the number and proximity of hydroelectric stations, providing a comprehensive evaluation of the factors involved.

Regarding the water quality, water temperature and dissolved oxygen have always been focal points of research, and the relationship between these two factors and biological responses has shown insignificant changes in the short term (Wu et al. 2023). Consequently, although the water quality here does not support a comprehensive quantitative analysis, the general trends discerned from qualitative analysis are of referential value. However, note that organisms gradually modify their dependency on environmental factors through adaptive evolution in the long term (Wu et al. 2023).

The construction of hydroelectric station results in the reserialization of rivers, altering the fluvial environment. Numerous studies have shown that the establishment of hydroelectric stations has a negative impact on aquatic organisms (Shakir et al. 2014; Chen et al. 2023). Our results also show that the detection rate of the species tends to decrease with the increase of hydroelectric stations (Table S2). For this result, we postulate that the construction of hydroelectric stations presents considerable risks to the survival and reproductive success of native fish species. Our results show that detection rates were high near the hydroelectric stations (Figure 5). Although our research does not differentiate between the upstream and downstream areas, both are suitable. In the upstream area, hydroelectric stations prevent the species from migrating; in the downstream area, the relatively shallow water and frequent water exchange promote the species' inhabitation (Bian et al. 2011; Li et al. 2022). Furthermore, the impact of hydroelectric stations is also determined by the structural and spatial attributes of the hydroelectric stations (Arantes et al. 2019), especially factors such as hydroelectric station height, reservoir size, and the longitudinal position within the river basin. Due to data limitations, this study does not delve into these aspects further, but future work will continue to explore these aspects.

### 4.3 Resource Conservation

As *Bangana. tungting* is classified as an endangered species, its survival status has garnered extensive attention. Research indicates that the survival of *B. tungting* is influenced by water temperature and dissolved oxygen concentrations. Consequently, it is crucial to monitor the water quality for the survival of *B. tungting*, and, in particular, dissolved oxygen at 4 mg/L is a critical threshold for the survival of *B. tungting* (Figure 5). Therefore, we further recommend implementing effective methods to increase dissolved oxygen concentrations for a higher survival rate. On the other hand, our study has also found that the number and proximity of hydroelectric stations significantly affect the survival of *B. tungting* (Figure 5). With the increasing number of hydroelectric stations and their proximity, numerous reports have highlighted the detrimental effects on aquatic organisms, particularly fish (Bednarek 2001; Jackson and Marmulla 2001). We propose that a similar phenomenon may be present and occurring in *B. tungting*. This is likely due to the hydroelectric station construction disrupting the habitats of *B. tungting*. Thus, in areas suitable for *B. tungting* habitation, it is advisable to minimize the number of hydroelectric stations, if possible, to minimize the potential effects for the species.

*Bangana tungting* is a freshwater fish species endemic to China, with significant ecological value and conservation importance. In this research, we successfully applied eDNA technology that minimizes ecological disruption to detect the existence of *B. tungting*. However, it is still challenging to protect *B. tungting* within a short time period. First, our understanding of the habitat and behavior of the species is incomplete and there is still a lack of data regarding its growth and development and survival rates in the wild. Second, it usually takes some time to restore the biological environment to the required condition, and therefore, the effects of conservation policy cannot be evaluated immediately. Third, since the species is less important than others in the region, unlawful fishing still happens and law enforcement needs to be strengthened. To further understand the survival status and biomass trends of *B. tungting*, we propose to continuously utilize eDNA technology for quantitative and long‐term tracking and monitoring of the DNA concentration of this species. Conservation sites will be established in areas where DNA is present, and activities such as fishing should be prohibited to avoid any impact on the resource recovery of *B. tungting*. By monitoring changes in DNA concentration, we can better assess the survival status of *B. tungting*. Additionally, we will investigate the interactions between various environmental factors and the survival and reproduction of *B. tungting*, aiming to identify more effective conservation measures.

The responsibility for conserving *B. tungting* lies not only with scientists but also with society as a whole. It is imperative to garner attention from all sectors of society toward the conservation of *B. tungting*, strengthening these efforts through legislation, regulations, and public education. Only with widespread societal participation can we truly achieve the effective conservation of *B. tungting*, allowing this unique freshwater species to thrive in our ecosystems.

## Author Contributions

**Lu Tian:** conceptualization (equal), data curation (equal), investigation (equal), writing – original draft (equal), writing – review and editing (equal). **Qianqian Wu:** conceptualization (equal), data curation (equal), formal analysis (equal), investigation (equal), visualization (equal), writing – original draft (equal), writing – review and editing (equal). **Li Zou:** data curation (equal), investigation (equal). **Jinxin Zhou:** data curation (equal), visualization (equal), writing – original draft (equal), writing – review and editing (equal). **Chongrui Wang:** data curation (equal). **Linmei Han:** data curation (equal). **Zaiquan Zhang:** investigation (equal). **Xing Xiang:** investigation (equal). **Mingqiu Liu:** investigation (equal). **Zhifeng Feng:** conceptualization (equal). **Zhonggui Xie:** conceptualization (equal), data curation (equal), investigation (equal). **Zhiqiang Liang:** conceptualization (equal), funding acquisition (lead), investigation (equal), writing – original draft (equal), writing – review and editing (equal).

## Conflicts of Interest

The authors declare no conflicts of interest.

## Supporting information


**Table S1.** Reference sequence information for the development of primers.
**Table S2.** Detection results at each sampling site, with supporting information indicating whether each sampling area is a protected area, whether there has been restocking in recent years, and showing the number of hydropower stations between each site, the distance to the nearest hydropower station at each site (+ means upstream; − means downstream), and environmental data from sampling in 2022.
**Table S3.** The importance of three correlated environmental factors. The three factors are treated as independent variables, with the species detection being a dependent variable.
**Figure S1.** The results of electrophoresis utilizing organizational samples. M denotes 100 bp “mark,” while the numbers 1–9 correspond to various species: target species *Bangana tungting* and non‐target species including *Bangana lippus* , *Bangana yunnanensis*, *Bangana tonkinensis* , *Bangana lemassoni* , *Bangana rendahli*, *Bangana dero*, *Bangana ariza*, and *Bangana decora*.

## Data Availability

The data supporting the findings of this study are available within the article [and/or] its Supporting Information.
