# Supplementary material for: Investigation on the Distribution of Bangana Tungting in Yuanshui Unique Fish Species National Aquatic Germplasm Resources Reserve Using Environmental DNA Technology
Source: Ecol Evol. 2024 Dec 12;14(12):e70626. doi: 10.1002/ece3.70626 (PMC11638367; doi:10.1002/ece3.70626)

Table S1

| **Species name** | **Accession No.** |
| --- | --- |
| *Bangana lippus* | JX074275.1 |
| *Bangana yunnanensis* | MG732849.1 |
| *Bangana tonkinensis* | GU086562.1 |
| *Bangana lemassoni* | GU086537.1 |
| *Bangana rendahli* | MG732850.1 |
| *Bangana dero* | MH973621.1 |
| *Bangana ariza* | MN273485.1 |
| *Bangana decora* | MG732851.1 |
| ******Bangana tungting* | NC027069.1 |

* Target species

Table S2

| **Sampling Area** | **ID** | **Distance from the nearest hydroelectric station (km)** | **Number**  **of hydroelectric station** | **Longitude / Latitude** | **Ct value** | | **Sept. 2022** | | | | **Core area/ Experimental area** | ***Bangana tungting***  **release site** |
| --- | --- | --- | --- | --- | --- | --- | --- | --- | --- | --- | --- | --- |
|  |  |  |  |  | **Sept. 2022** | **May 2023** | **Tem (℃)** | **DO (mg/L)** | **NH3-N (mg/L)** | **pH** |  |  |
| TK | TK1 | +2 | 3 | 109.661659, 27.13358 |  | 38.01 | 28.0 | 11.82 | 0.06 | 8.82 | Core area | N |
|  | TK2 | +1.55 | 3 | 109.649892, 27.157192 |  |  |  |  |  |  |  |  |
|  | TK3 | +0.5 | 3 | 109.658084, 27.154893 |  |  |  |  |  |  |  |  |
|  | TK4 | +0.1 | 3 | 109.653054, 27.157915 |  |  |  |  |  |  |  |  |
|  | TK5 | –0.2 | 4 | 109.662827, 27.164215 |  |  |  |  |  |  |  |  |
|  | TK6 | –1.2 | 4 | 109.668576, 27.182469 | 36.49 |  |  |  |  |  |  |  |
|  | TK7 | –1.5 | 4 | 109.671738, 27.19121 |  |  |  |  |  |  |  |  |
|  | TK8 | –2.5 | 4 | 109.675188, 27.191467 |  |  |  |  |  |  |  |  |
| WS | WS1 | +40 | 4 | 109.822079, 27.19905 |  |  | 29.6 | 7.04 | 0.07 | 8.44 | Core area | Y |
|  | WS2 | +40 | 4 | 109.827541, 27.208561 |  |  |  |  |  |  |  |  |
|  | WS3 | +40 | 4 | 109.819779, 27.196608 |  |  |  |  |  |  |  |  |
|  | WS4 | +40 | 4 | 109.82596, 27.200721 |  |  |  |  |  |  |  |  |
| BYD | BYD1 | +1.5 | 4 | 109.910778, 27.147211 |  | 36.75 | 29.0 | 9.46 | 0.06 | 8.75 | Core area | Y |
|  | BYD2 | +1.5 | 4 | 109.910005, 27.145651 | 36.58 |  |  |  |  |  |  |  |
|  | BYD3 | +1.5 | 5 | 109.915539, 27.144687 |  |  |  |  |  |  |  |  |
|  | BYD4 | +1.5 | 5 | 109.917156, 27.149091 |  |  |  |  |  |  |  |  |
| DWT | DWT1 | –0.5 | 5 | 109.979444, 27.133194 |  |  | 30.1 | 3.87 | 0.07 | 7.1 | Core area | N |
|  | DWT2 | –2 | 5 | 109.984475, 27.130397 |  |  |  |  |  |  |  |  |
|  | DWT3 | –2 | 5 | 109.982858, 27.13329 |  | 37.61, 36.36，35.96 |  |  |  |  |  |  |
|  | DWT4 | –3 | 5 | 109.996063, 27.128371 |  |  |  |  |  |  |  |  |
| LBW | LBW1 | +5 | 11 | 110.03027,27.124577 |  |  | 28.5 | 6.0 | 0.06 | 8.33 | Core area | N |
|  | LBW2 | +5 | 11 | 110.030989, 27.140138 |  |  |  |  |  |  |  |  |
|  | LBW3 | +5 | 11 | 110.015897, 27.144896 |  |  |  |  |  |  |  |  |
|  | LBW4 | +5 | 11 | 110.013023, 27.145153 |  |  |  |  |  |  |  |  |
| TOW | TOW1 | +0.2 | 11 | 110.307352, 27.58106 |  | Missing data | 29.5 | 6.54 | 0.15 | 8.82 | Experimental area | N |
|  | TOW2 | +0.15 | 11 | 110.300957, 27.586759 |  |  |  |  |  |  |  |  |
|  | TOW3 | –0.15 | 12 | 110.29377, 27.595532 |  |  |  |  |  |  |  |  |
|  | TOW4 | –0.2 | 12 | 110.28953, 27.602191 |  |  |  |  |  |  |  |  |
| XRW | XRW1 | +0.5 | 12 | 110.377995, 27.752164 |  |  | 29.5 | 5.18 | 0.08 | 8.61 | Experimental area | N |
|  | XRW2 | +0.1 | 12 | 110.376702, 27.754593 | 37.71 |  |  |  |  |  |  |  |
|  | XRW3 | –0.2 | 13 | 110.351837, 27.7638 |  |  |  |  |  |  |  |  |
|  | XRW4 | –0.3 | 13 | 110.345728, 27.771599 |  |  |  |  |  |  |  |  |
| DJK | DJK1 | +0.5 | 13 | 110.415293, 27.88571 |  |  | 29.6 | 8.84 | 0.05 | 8.48 | Experimental area | N |
|  | DJK2 | +0.1 | 13 | 110.424995, 27.891457 |  |  |  |  |  |  |  |  |
|  | DJK3 | –0.2 | 14 | 110.399411, 27.884816 |  |  |  |  |  |  |  |  |
|  | DJK4 | –1 | 14 | 110.395889, 27.879899 |  |  |  |  |  |  |  |  |
| TW | TW1 | +0.5 | 14 | 110.284212, 27.981422 |  |  | 30.3 | 7.84 | 0.05 | 8.58 | Experimental area | Y |
|  | TW2 | +0.5 | 14 | 110.28335,27.988057 |  |  |  |  |  |  |  |  |
|  | TW3 | –0.6 | 15 | 110.169157, 27.959823 |  |  |  |  |  |  |  |  |
|  | TW4 | –0.6 | 15 | 110.194597, 27.964641 |  |  |  |  |  |  |  |  |
| GYDZ | GYDZ1 | +0.1 | 3 | 110.029435, 26.950389 |  | 49.13 | 30.0 | 3.84 | 0.16 | 7.5 | Core area | N |
|  | GYDZ2 | +0.1 | 3 | 110.029129, 26.950501 |  |  |  |  |  |  |  |  |
|  | GYDZ3 | –0.05 | 4 | 110.027135, 26.953126 |  |  |  |  |  |  |  |  |
|  | GYDZ4 | –0.1 | 4 | 110.025698, 26.953255 |  |  |  |  |  |  |  |  |
| GY | GY1 | +3 | 4 | 110.033315, 26.957924 | 39.09 |  | 30.2 | 4.61 | 0.04 | 7.54 | Core area | Y |
|  | GY2 | +4 | 4 | 110.034357, 26.957586 | 38.54 |  |  |  |  |  |  |  |
|  | GY3 | +5 | 4 | 110.037268, 26.965072 |  |  |  |  |  |  |  |  |
|  | GY4 | +6 | 4 | 110.0384, 26.965072 |  |  |  |  |  |  |  |  |
|  | GY5 | +5 | 4 | 110.032363, 26.983183 |  |  |  |  |  |  |  |  |
|  | GY6 | +4 | 4 | 110.026327, 26.984358 |  |  |  |  |  |  |  |  |
|  | GY7 | +3 | 4 | 110.014828, 26.997492 |  |  |  |  |  |  |  |  |
|  | GY8 | +2 | 4 | 110.01296, 26.998007 | 38.62 |  |  |  |  |  |  |  |
| HMX | HMX1 | –0.6 | 5 | 109.998084, 27.073756 |  |  | 29.2 | 6.47 | 0.05 | 8.13 | Core area | N |
|  | HMX2 | –0.8 | 5 | 110.001821, 27.081733 |  |  |  |  |  |  |  |  |
|  | HMX3 | –0.8 | 5 | 109.997796, 27.076844 |  |  |  |  |  |  |  |  |
|  | HMX4 | –1 | 5 | 109.997509, 27.070153 |  |  |  |  |  |  |  |  |
| YLW | YLW1 | +0.2 | 5 | 110.000096, 27.102832 |  |  | 30.4 | 5.84 | 0.09 | 7.17 | Core area | N |
|  | YLW2 | +0.2 | 5 | 109.998371, 27.102832 | 36.91, 38.83 |  |  |  |  |  |  |  |
|  | YLW3 | –0.1 | 6 | 110.006492, 27.10661 |  |  |  |  |  |  |  |  |
|  | YLW4 | –0.1 | 6 | 110.009169, 27.107109 | 36.72, 38.26, 38.26 |  |  |  |  |  |  |  |

Table S3

| **Environmental factors** | **Importance** | **Sign** |
| --- | --- | --- |
| Dissolved oxygen | 0.7227723 | 1 |
| pH | 0.7135679 | –1 |
| Water temperature | 0.4385099 | 1 |

Fig S1.


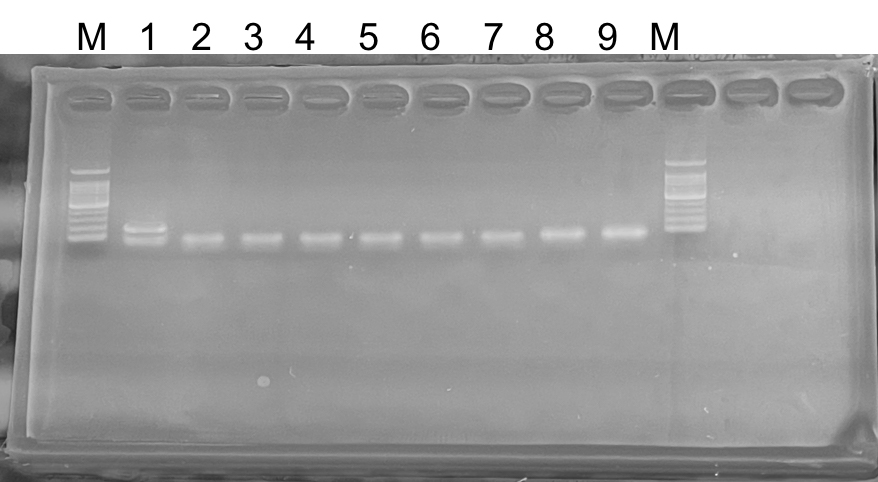

Supplement: Supplementary file 1 — Table S1. Reference sequence information for the development of primers. Table S2. Detection results at each sampling site, with supporting information indicating whether each sampling area is a protected area, whether there has been restocking in recent years, and showing the number of hydropower stations between each site, the distance to the nearest hydropower station at each site (+ means upstream; − means downstream), and environmental data from sampling in 2022. Table S3. The importance of three correlated environmental factors. The three factors are treated as independent variables, with the species detection being a dependent variable. Figure S1. The results of electrophoresis utilizing organizational samples. M denotes 100 bp “mark,” while the numbers 1–9 correspond to various species: target species Bangana tungting and non‐target species including Bangana lippus , Bangana yunnanensis, Bangana tonkinensis , Bangana lemassoni , Bangana rendahli, Bangana dero, Bangana ariza, and Bangana decora. [file ECE3-14-e70626-s001.docx]
